# Optimization of Energy Efficiency, Operation Costs, Carbon Footprint and Ecological Footprint with Reverse Osmosis Membranes in Seawater Desalination Plants

**DOI:** 10.3390/membranes11100781

**Published:** 2021-10-12

**Authors:** Federico Leon, Alejandro Ramos, Sebastian O. Perez-Baez

**Affiliations:** Departamento de Ingeniería de Procesos, Universidad de Las Palmas de Gran Canaria, 35017 Las Palmas de Gran Canaria, Spain; alejandro.ramos@ulpgc.es (A.R.); sebastianovidio.perez@ulpgc.es (S.O.P.-B.)

**Keywords:** energy efficiency, reverse osmosis, membranes, desalination

## Abstract

This article shows the optimization of the reverse osmosis process in seawater desalination plants, taking the example of the Canary Islands, where there are more than 320 units of different sizes, both private and public. The objective is to improve the energy efficiency of the system in order to save on operation costs as well as reduce the carbon and ecological footprints. Reverse osmosis membranes with higher surface area have lower energy consumption, as well as energy recovery systems to recover the brine pressure and introduce it in the system. Accounting for the operation, maintenance and handling of the membranes is also important in energy savings, in order to improve the energy efficiency. The energy consumption depends on the permeate water quality required and the model of the reverse osmosis membrane installed in the seawater desalination plant, as it is shown in this study.

## 1. Introduction

Seawater desalination in water treatment plants has evolved considerably in the last five decades, in which the desalination process and its technology have changed and become more and more profitable and efficient. Initially, the water desalination process was a thermal process, but it has been changing with scientific technological advances towards a process of reverse osmosis, which dominates the current market [1,2,3,4,5].

Following the state of the art in water desalination and the evolution of this process not only at the regional Canary level but also at national and international levels, there are now different desalination processes, such as Vapor Compression (VC), Multi-Effect Distillation (MSF), Multi-Stage Distillation (MED) and reverse osmosis, which currently account for 65% of all the processes used around the world [4,5,6,7].

The main objective is to study the improvements in seawater desalination, based on the reduction of energy consumption in the production of fresh water. Consequently, reverse osmosis is the most suitable process due to its lower energy consumption per cubic meter of water produced, and therefore it occupies a privileged position in the sector. So far, in the 21st century, research efforts in water desalination have focused on advances in reverse osmosis membranes, with higher surface area and lower energy consumption, as well as energy recovery systems to recover the brine pressure and introduce it in the system, reducing the energy consumption of the desalination process [8,9,10].

The operation, maintenance and handling of the membranes have been studied in detail, due to their importance in energy savings, detailing how to optimize all the processes in which they are involved to improve energy efficiency [7].

In the same way, we analyze data from the different seawater desalination plants we visited, obtaining data on thousands of hours of operation in many cases. We have developed techniques to improve the energy efficiency of seawater desalination membranes in strict compliance with the water quality parameters established by national and international regulations, or even by organizations such as the World Health Organization [11,12,13,14,15,16,17,18,19].

To carry out a general cost analysis of the components or elements of the plant and their operation, it is necessary to determine the direct costs, indirect costs and other considerable expenses for this purpose [20,21,22,23,24].

Among the direct costs, we can highlight the acquisition cost of the elements, both initial and replacement, and among the most significant expenses are those related to the initial capital investment, operation and maintenance [25,26,27].

## 2. Materials and Methods

As stated earlier, energy consumption depends on the permeate water quality required and the reverse osmosis membrane model installed in the desalination plant. Therefore, we developed a methodology, in the following equations, to calculate the permeate quality–cost ratio [15,16,17,18,19].

C_EE_ = f_1_ × E_E_ = f_1_ × E_b/_µ_e_ = f_1_ × E_h/_(µ_b_ × µ_e_) = f_1_ × ρ × g × h_b_/(c × µ_b_ × µ_e_) (1)

Q = Q′/c (2)

P_h_ = ρ × g × Q × h_b_ = ρ × g × Q’/c × h_b_ (3)

E_h_ = P_h_/Q′ = ρ × g × h_b_/c (4)

E_B_ = E_h_/µ_b_ (5)

E_E_ = E_B_/µ_e_ (6)

h_b(año 1–5)_ = (1.2 × t_m_ + 0.6) + h_b(año 0)_ (7)

c: Plant recovery.

ρ: Fluid density (1000 kg/m^3^ for water).

g: Acceleration of gravity (generally adopted: 9.81 m/s^2^).

h_b_: Manometric pump height (m).

C_EE_: Cost of electricity per cubic meter of water produced.

f_1_: Factor about the price of the electric energy consumed EUR/kWh.

P_h_: Hydraulic power transmitted to water.

Pe: Power consumption.

Q′: Permeate flow rate.

Q: Feed rate.

µ_b_: High pressure pump performance.

µ_e_: Electrical performance of the high-pressure pump.

P_B_: Pump power.

E_E_: Electrical energy consumed per cubic meter of water produced.

E_B_: Total energy consumed by the pump per cubic meter of produced water.

E_h_: Hydraulic energy per cubic meter of produced water.

δ_E_: Electrical losses.

δ_h_: Hydraulic losses.

δ_m_: Mechanical pump losses.

h_b_: Pump head (m).

t_m_: Age of the membrane.

**Figure 1:**
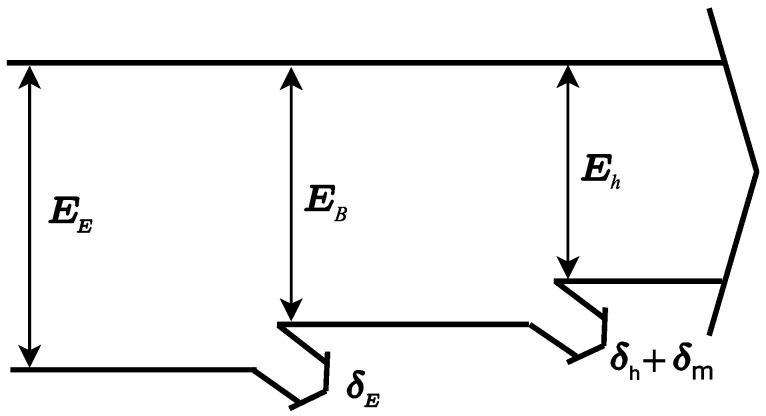
Energy block diagram.

Figure 1 below shows the energy block diagram, which includes the electrical, hydraulic and mechanical pressure losses that occur in the process.

### General Analysis of Element and Operation Costs

In this sense, and as a guide, according to data from a construction company of desalination plants in Gran Canaria with more than 100 references in the market, it should be noted that the cost of the membranes in a seawater desalination plant represents approximately 13% of the total investment in the facility’s equipment. The rest of the components (high-pressure pump, booster pump, pressure pipes, pre-treatment, etc.) represent 87% of the total amount, not including industrial profit and before taxes [1,2,28].

**Figure 2:**
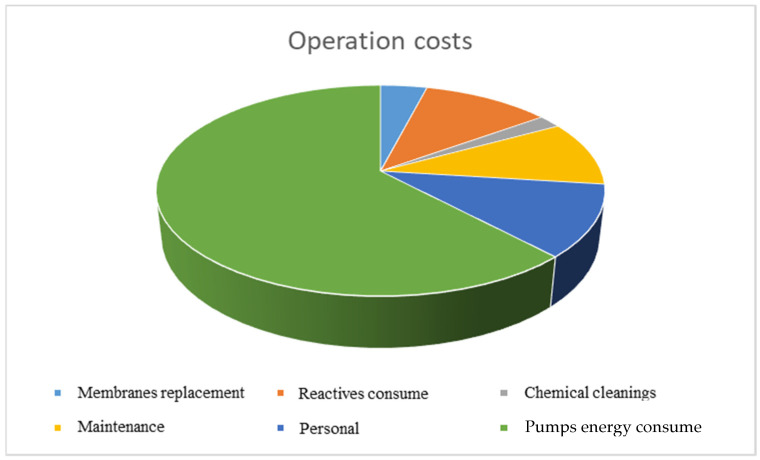
Operation costs.

Table 1 and Figure 2 show all the significant variables that affect operating costs per cubic meter of water produced [3,4,5,6,7].

In this sense, it is demonstrated that the cost of energy consumption in the pumps and mainly in the high-pressure pump is by far the most significant of a seawater desalination plant, and we can reduce it considerably with the introduction of last-generation reverse osmosis membranes, which were confirmed to be suitable through the same through-plant pilots [29,30].

If the membranes are not replaced, an action that has the lowest cost of those studied, this will have a negative impact with a considerable increase in the energy consumption of the high-pressure pump, which very significantly affects the cost per m3 of water produced, as discussed below [31,32,33,34].

**Figure 3:**
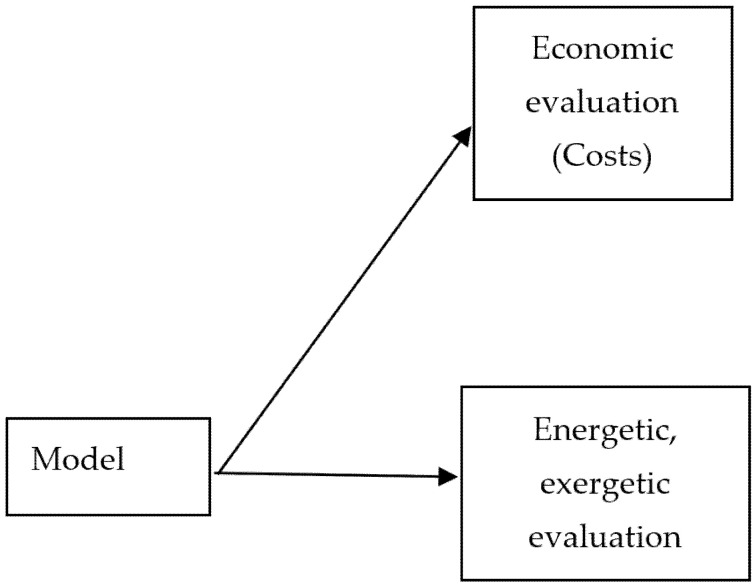
Energy, exergy and economic block diagram.

**Figure 4:**
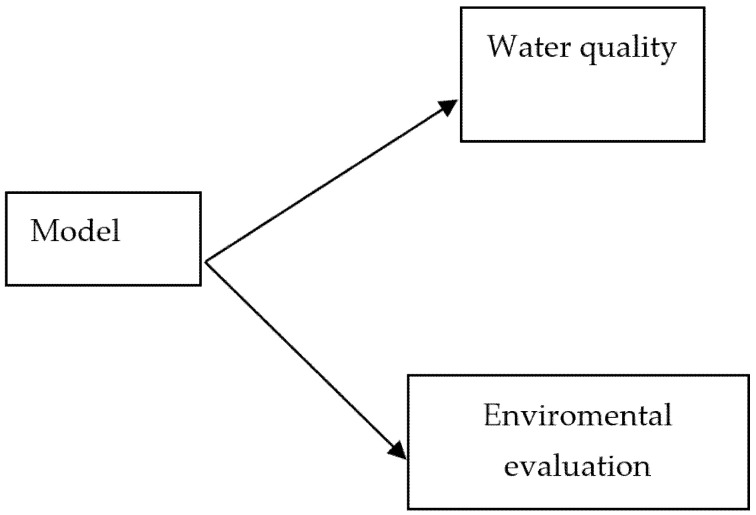
Environmental and water quality block diagram.

In Figure 3 and Figure 4, the most important issues of this model are represented, which are the costs, energy consumption, water quality and environment.

A reduction in energy consumption will have a direct impact on environmental improvement and we study this through the carbon footprint produced by these desalination plants and their ecological footprint, with the latter as a future line of action. The corresponding diagram according to Figure 4 is shown below.

To produce a quantity of water from a reverse osmosis plant, a quantity of electrical energy must be consumed, and to generate this energy in a conventional electrical network, emissions in the form of greenhouse gases are emitted.

The magnitude of these emissions depends on the set of technologies that make up the energy generation system of the electrical network to which the water production plant is connected. The energy produced by this set is often referred to as the energy mix, which tends to depend largely on the territory and energy policy [3,4].

In relation to territorial dependence, electricity networks generally have energy mixes that cause higher greenhouse gas emissions, as they generally have systems based on lower performance technologies. These electrical energy production technologies can mainly be classified as two types: Conventional and renewable [3].

Within the conventional technologies, which have a direct impact on the carbon footprint of the installations, several can be considered: Diesel engines, gas turbines, combined cycles and steam turbines, which generally have different performances and quantities of emissions. On the other hand, there are technologies based on renewable energies, such as solar photovoltaic, wind, waves, etc. [4,5].

Therefore, in order to reduce greenhouse gas emissions, it is possible to propose the generation of electrical energy necessary for water production in the same facility through hybrid energy systems. These hybrid energy systems can be composed of several types of technologies, in which the largest amount of energy from renewable sources tends to be integrated with the support of an energy storage system or conventional technology such as a diesel engine [3].

Therefore, a methodology can be proposed to achieve the stable operation of a high-efficiency diesel engine with a small integrated autonomous diesel engine and a photovoltaic solar energy generating system to power a reverse osmosis plant, thus reducing the greenhouse gas emissions associated with water production. This application would be very useful in hotel complexes, private facilities, industries, isolated areas, etc. [3].

For the specific case of seawater desalination plants in the Canary Islands, with regard to the production of seawater desalination plants, the following permeate flows can be confirmed: Gran Canaria (220,870 m^3^/d), Tenerife (106,034 m^3^/d), Fuerteventura (90,755 m^3^/d) and Lanzarote (87,480 m^3^/d). These produce a significant carbon footprint with respect to the overall footprint of each island, especially on Fuerteventura and Lanzarote. In this sense, renewable energies can make a great contribution, mainly through wind and solar photovoltaics. For example, Fuerteventura and Lanzarote are windy islands with high solar radiation all year round, which also have large areas of flat land suitable for these installations. These installations could be for the energy consumption of public desalination plants, or for those that are private, which are normally smaller and can also be self-supplied with renewable energies and a diesel engine for the security of the electricity supply at all times without resorting to the island network, as may be the case of hotels or isolated areas where the electricity network does not reach. In Gran Canaria and Tenerife, it is also possible to implement this, although the orography is more complicated throughout the year in the coastal areas where the seawater desalination plants are located, as the solar radiation and the winds are quite significant, especially in the months between June and September with sunnier days and trade winds. Therefore, the possibility of introducing renewable energies for the supply of electricity to seawater desalination plants in the Canary Islands is studied in order to reduce the carbon footprint and the ecological footprint of the sector, due to the considerable influence of the whole archipelago.

Similarly, to calculate the ecological footprint, we follow previous methodology [11,12,13,14], which is expressed in Table 2.

## 3. Results

Taking into account these parameters, the typical production of a seawater plant of 100,000 m^3^/d, Equation (7) explained above and the reverse osmosis membrane software, we obtain the common results presented in Table 3, Table 4 and Table 5.

In Table 3, there is a pressure difference essentially every year, due to the age of the membranes. At start up, in year 0, the elements are new so they need less feed pressure than in years 1 to 5. This is because fouling and scaling could damage the membranes little by little, and consequently, the feed pressure increases every year. This shows that the pressure measured in year 1 grows more in the first year, and from year 2, it is constant at 1.2 bar.

**Figure 5:**
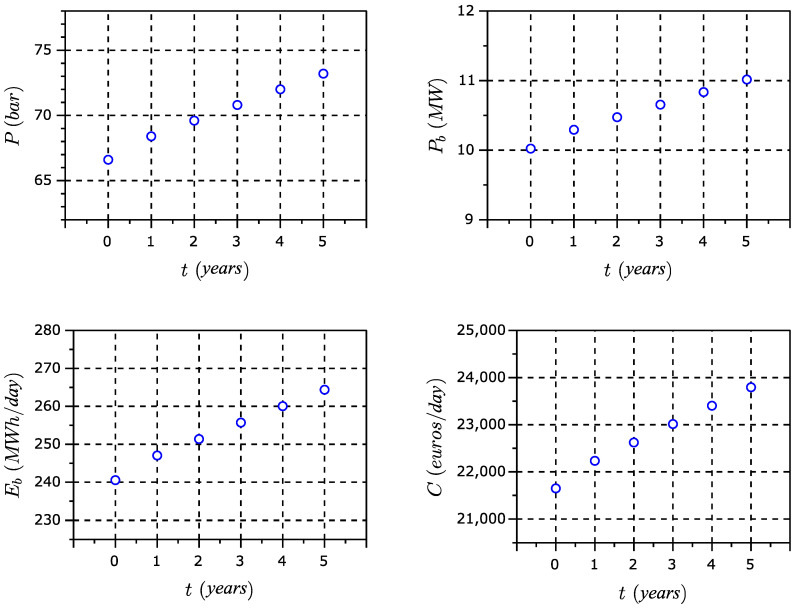
Pressure, power, energy and cost.

Consequently, one can observe from Figure 5 that the pressure varies over 5 years without replacing the membranes, whereas the energy consumption of the pump increases accordingly.

In Table 4, feed temperature is low (17 °C), and due to this, the feed pressure is higher than in Table 5 where the feed temperature is high (27 °C). At start up, the feed pressure is 6–7 bars higher at 17 °C than at 27 °C. After 5 years, without replacement, the pressure difference is even higher between the minimum and maximum temperature, at around 9–10 bars.

In Table 6, we show the existing seawater desalination plants in the Canary Islands, including consumption, and the introduction of renewable energies.

Table 7 shows the existing seawater desalination plants in the Canary Islands including the carbon and ecological footprints.

**Figure 6:**
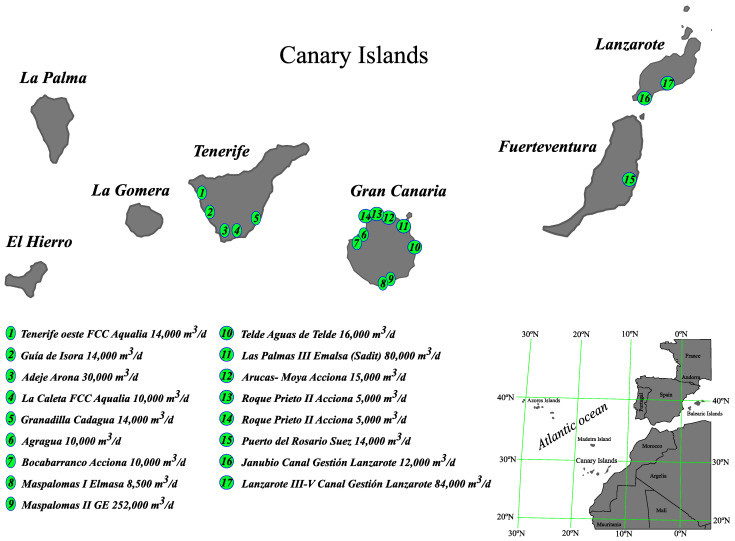
Most significant seawater desalination plants (2019).

Figure 6 shows the most significant plants in the Canary Islands, in terms of size, that produce the largest share of the ecological footprint mentioned above. Moreover, the positions of the RO desalination plants are shown on the map, including the permeate flow of each one in the picture.

Considering the type of specific environmental impact indicators [10], the results are classified according to the non-renewable technology and island in Table 8 (2019).

Table 9 presents the above values per MW of installed power on each island.

Similarly, we can calculate the CO_2_ footprint per MWh taking into account the thermal consumption by technology and island in Table 10 and Table 11.

## 4. Conclusions

The most important conclusions obtained from this study are the following:

- By reducing the operation costs outlined in this article, it is possible to improve the energy efficiency of the system.
- To reduce the carbon footprint and ecological footprint, the energy consumption needs to be decreased.
- There are different results regarding the optimization of energy efficiency and environmental footprints.
- These conclusions of the study may serve as a tool for the decision-making processes related to improving energy efficiency in seawater reverse osmosis plants.
- The main objective was to study the improvements in seawater desalination based on the reduction of energy consumption in the production of fresh water.
- Reverse osmosis is the most suitable process due to its lower energy consumption per cubic meter of water produced.
- Reverse osmosis membranes with higher surface area have lower energy consumption, as well as energy recovery systems to recover the brine pressure and introduce it in the system, reducing the energy consumption of the desalination process.
- Considering the operation, maintenance and handling of the membranes is also important in energy savings, in order to improve energy efficiency.
- Energy consumption depends on the permeate water quality required and the model of the reverse osmosis membrane installed in the desalination plant.

## Figures and Tables

**Table 1:** Operation costs.

Operation Cost	Nomenclature	Percentage (%)
Membranes replacement	Cm	4
Reagents consumption	Cr	11
Chemical cleanings	Cq	2
Maintenance	Cm	10
Staff	Cp	11
Pumps energy consumption	Ce	62

**Table 2:** Average and equivalent CO_2_ absorption per hectare of the different surfaces of planet Earth. Surface area equivalence factors.

Category Surfice	ABS. Average (tCO_2_/ha/Year)	Surface (Millions ha)	%	ABS. Hectarea Equivalent (tCO_2_/ha/Year)	Equivalence Factor (fi)
Forests	19.35	3858.10	7.56	1.46	9.66
Crops	8.09	1958.32	3.84	0.31	4.04
Medows and pastures	2.44	3363.72	6.59	0.16	1.22
Oceans, seas, etc…	0.10	36,010.00	70.60	0.07	0.05
Deserts	0.00	3600.00	7.06	0.00	0.00
Others	0.00	2217.06	4.35	0.00	0.00
Total Surface		51,007.20		2.00	1.00

**Table 3:** Pressure increases without membrane replacement at 22 °C.

Year	Pressure (bar)	Power (kW)	Energy (kWh/d)	Cost (€/d)
0	66.6	10,023.5	240,564.9	21,625.6
1	68.4	10,294.4	247,066.7	22,210.1
2	69.6	10,475.0	251,401.2	22,599.7
3	70.8	10,655.7	255,735.7	22,989.4
4	72.0	10,836.3	260,070.2	23,379.0
5	73.2	11,016.9	264,404.7	23,768.7

**Table 4:** Pressure increases without membrane replacement at 17 °C.

Year	Pressure (bar)	Power (kW)	Energy (kWh/d)	Cost (€/d)
0	69.5	10,460.0	251,039.9	22,567.2
1	72.6	10,926.5	262,237.5	23,573.8
2	74.4	11,197.4	268,739.2	24,158.3
3	76.0	11,438.3	274,518.5	24,677.8
4	77.5	11,664.1	279,936.7	25,164.9
5	78.9	11,874.8	284,993.6	25,619.5

**Table 5:** Pressure increases without membrane replacement at 27 °C.

Year	Pressure (bar)	Power (kW)	Energy (kWh/d)	Cost (€/d)
0	62.9	9466.6	227,200.2	20,424.1
1	65.1	9797.7	235,146.8	21,138.5
2	66.3	9978.3	239,481.3	21,528.2
3	67.3	10,128.9	243,093.4	21,852.9
4	68.3	10,279.4	246,705.5	22,177.6
5	69.2	10,414.9	249,956.4	22,469.8

**Table 6:** Existing seawater desalination plants in the Canary Islands, consumption and solution of renewable energies. Source: FCCA 2013, REE 2020 and own elaboration.

Name of the Plant	Production (m^3^/d)	Consume (kWh/m^3^)	Island	Habitants per Plant	Renewable Solution
Cercado de Don Andrés	200	3.5	Lanzarote	Irrigation	Photovoltaic
Lanzarote III 1	10,000	3.5	Lanzarote	10,541	Wind
Lanzarote III 2	5000	3.5	Lanzarote	5271	Wind
Lanzarote III 3	5000	3.5	Lanzarote	5271	Wind
Lanzarote IV	20,000	3.5	Lanzarote	21,083	Wind
Lanzarote V	18,000	2.4	Lanzarote	18,975	Wind
Aeropuerto	700	3.04	Lanzarote	18,327	Photovoltaic
Agua Park	30	3.04	Lanzarote	500	Photovoltaic
Apartamentos Ficus	60	3.5	Lanzarote	120	Photovoltaic
Apartamentos Puerto Tahiche	150	3.5	Lanzarote	300	Photovoltaic
Apartamentos Trebol	80	3.5	Lanzarote	160	Photovoltaic
Ercros	2500	3.5	Lanzarote	11,057	Wind
Ercros	2200	3.5	Lanzarote	9731	Wind
Famara	350	3.5	Lanzarote	700	Photovoltaic
Hotel Golf y Mar	90	3.5	Lanzarote	180	Photovoltaic
Hotel Gran Meliá Salinas	400	2.61	Lanzarote	800	Photovoltaic
Hotel Playa Verde	250	3.5	Lanzarote	500	Photovoltaic
Hotel Teguise Playa	250	3.5	Lanzarote	500	Photovoltaic
La Galea	150	3.04	Lanzarote	300	Photovoltaic
Lanzarote Beach Club II	70	3.04	Lanzarote	140	Photovoltaic
Las Arenas. Costa Teguise	80	3.04	Lanzarote	160	Photovoltaic
Playa Roca	250	3.04	Lanzarote	500	Photovoltaic
Apartamentos Don Paco Castilla	320	2.61	Lanzarote	640	Photovoltaic
Apartamentos Sol Lanzarote	350	2.61	Lanzarote	700	Photovoltaic
Cdad Apartamentos CAMP		2.61	Lanzarote	Tourism	Photovoltaic
Holiday Land S.A.	3000	3.5	Lanzarote	6000	Wind
Hotel Fariones Playa	500	3.5	Lanzarote	1000	Photovoltaic
Hotel Playa Azul	300	3.5	Lanzarote	600	Photovoltaic
Hoteles Canarios S.A.		3.5	Lanzarote	Tourism	Photovoltaic
Iberhotel		3.5	Lanzarote	Tourism	Photovoltaic
Zorilla	40	3.04	Lanzarote	80	Photovoltaic
Hotel Jameos Playa	336	2.61	Lanzarote	672	Photovoltaic
La Santa Sport I	250	3.5	Lanzarote	500	Photovoltaic
La Santa Sport II	250	3.5	Lanzarote	500	Photovoltaic
Ria La Santa	400	3.5	Lanzarote	800	Photovoltaic
Apartamentos Son Boy Family Suites	500	3.04	Lanzarote	1000	Photovoltaic
Bungalows Atlantic Gardens		3.5	Lanzarote	Tourism	Photovoltaic
Costa los Limones S.A.	350	3.5	Lanzarote	700	Photovoltaic
Hotel Corbeta		3.5	Lanzarote	Tourism	Photovoltaic
Hotel Costa Calero	324	3.04	Lanzarote	642	Photovoltaic
Marina Rubicón	300	3.04	Lanzarote	600	Photovoltaic
Hotel Paradise Island	300	3.04	Lanzarote	600	Photovoltaic
Hotel Princesa Yaiza	500	3.04	Lanzarote	1000	Photovoltaic
Hotel Rubicón Palace	450	3.04	Lanzarote	900	Photovoltaic
Inalsa Sur 1	600	3.5	Lanzarote	1859	Photovoltaic
Inalsa Sur 2	1200	3.5	Lanzarote	3718	Wind
Inalsa Sur 3	3000	3.5	Lanzarote	9294	Wind
Janubio		3.04	Lanzarote	Tourism	Photovoltaic
Lanzasur Club	200	3.04	Lanzarote	400	Photovoltaic
Playa Blanca S.A.		3.5	Lanzarote	Tourism	Photovoltaic
Club Lanzarote	4500	3.5	Lanzarote	9000	Wind
Apartamentos Moromar	250	3.5	Lanzarote	500	Photovoltaic
Gea Fonds Numero Uno Lanzarote S.A.		3.5	Lanzarote	Tourism	Photovoltaic
Grupo Rosa	1000	3.5	Lanzarote	2000	Wind
Hipotels	300	3.5	Lanzarote	600	Photovoltaic
Hotel Corona	300	3.5	Lanzarote	600	Photovoltaic
Hotel Costa Calero S.L.	300	3.04	Lanzarote	600	Photovoltaic
Hotel Sunbou	500	3.04	Lanzarote	1000	Photovoltaic
Isla Lobos	100	3.04	Lanzarote	200	Photovoltaic
Leas Hotel S.A.		3.5	Lanzarote	Tourism	Photovoltaic
Niels Prahm		3.5	Lanzarote	Tourism	Photovoltaic
Occidental Hotel Oasis	250	3.04	Lanzarote	500	Photovoltaic
Playa Flamingo	200	3.04	Lanzarote	400	Photovoltaic
Tjaereborg Timesharing, S.A.	500	3.04	Lanzarote	1000	Photovoltaic
Empresa Mixta de Aguas de Antigua, S.L.	4800	3.04	Fuerteventura	11,948	Wind
Grupo Turístico Barceló, S.L.	240	3.5	Fuerteventura	480	Photovoltaic
Aguas Cristóbal Franquis, S.L.	1200	3.5	Fuerteventura	2400	Wind
Anjoca Canarias, S.A.	3000	3.5	Fuerteventura	6000	Wind
Ramiterra, S.L.	3000	3.04	Fuerteventura	6000	Wind
Inver Canary Dos, S.L.	300	3.04	Fuerteventura	600	Photovoltaic
Suministros de Agua de La Oliva, S.A.	9000	3.04	Fuerteventura	17,920	Wind
Consorcio Abastecimiento de Aguas a Fuerteventura	4000	3.04	Fuerteventura	7964	Wind
Parque de Ocio y Cultura (BAKU) 1	300	3.04	Fuerteventura	600	Photovoltaic
Parque de Ocio y Cultura (BAKU) 2	90	3.04	Fuerteventura	180	Photovoltaic
RIU Palace Tres Islas	100	3.5	Fuerteventura	200	Photovoltaic
RIU Oliva Beach	400	3.5	Fuerteventura	800	Photovoltaic
Nombredo, S.L.	500	3.5	Fuerteventura	1000	Photovoltaic
Consorcio Abastecimiento de Aguas a Fuerteventura	4400	3.5	Fuerteventura	20,539	Wind
Puertito de la Cruz	60	3.5	Fuerteventura	120	Photovoltaic
Vinamar, S.A.	3600	3.5	Fuerteventura	7200	Wind
Fuercan, S.L. Cañada del Rio I	2000	3.5	Fuerteventura	4000	Wind
Fuercan, S.L. Cañada del Rio II	1000	3.04	Fuerteventura	2000	Wind
Fuercan, S.L. Cañada del Rio III	2000	3.04	Fuerteventura	4000	Wind
Club Aldiana	200	3.5	Fuerteventura	400	Photovoltaic
Erwin Sick	30	3.5	Fuerteventura	60	Photovoltaic
Esquinzo Urbanización II	1200	3.5	Fuerteventura	2400	Wind
Esquinzo Urbanización III	1200	3.5	Fuerteventura	2400	Wind
Hotel Sol Élite Los Gorriones 1	400	3.5	Fuerteventura	800	Photovoltaic
Hotel Sol Élite Los Gorriones 2	400	3.5	Fuerteventura	800	Photovoltaic
Stella Canaris I	300	3.5	Fuerteventura	600	Photovoltaic
Stella Canaris II	300	3.5	Fuerteventura	600	Photovoltaic
Stella Canaris III	250	3.5	Fuerteventura	500	Photovoltaic
Hotel H 10 Playa Esmeralda.	250	3.5	Fuerteventura	500	Photovoltaic
Hotel “Club Paraíso Playa”	300	3.5	Fuerteventura	600	Photovoltaic
Urbanización Costa Calma.	110	3.5	Fuerteventura	220	Photovoltaic
Urbanización Tierra Dorada.	120	3.5	Fuerteventura	240	Photovoltaic
Zoo-Parque La Lajita.	1300	3.5	Fuerteventura	500	Wind
Apartamentos Esmeralda Maris	120	3.5	Fuerteventura	240	Photovoltaic
Hotel H10 Tindaya	280	3.5	Fuerteventura	560	Photovoltaic
Aparthotels Morasol	80	3.5	Fuerteventura	160	Photovoltaic
Consorcio Abastecimiento de Aguas a Fuerteventura	36,500	3.5	Fuerteventura	39,382	Wind
Aeropuerto	500	3.5	Fuerteventura	15,439	Photovoltaic
GranTarajal	4000	3.5	Fuerteventura	14,791	Wind
Sotavento, S.A.	2925	3.5	Fuerteventura	5850	Wind
Arucas-Moya I	10,000	3.5	Gran Canaria	45,419	Wind
Granja experimental	500	3.5	Gran Canaria	Irrigation	Photovoltaic
Granja experimental	500	3.5	Gran Canaria	Irrigation	Photovoltaic
Comunidad Fuentes de Quintanilla	800	3.04	Gran Canaria	Irrigation	Photovoltaic
Granja experimental	500	3.5	Gran Canaria	Irrigation	Photovoltaic
Gáldar-Agaete I	3000	3.5	Gran Canaria	16,199	Wind
Gáldar II	7000	3.04	Gran Canaria	37,799	Wind
Agragua	15,000	3.5	Gran Canaria	Irrigation	Wind
Guía I	5000	3.5	Gran Canaria	6962	Wind
Guía II	5000	2.61	Gran Canaria	6962	Wind
Félix Santiago Melián	5000	2.61	Gran Canaria	Irrigation	Wind
Las Palmas III	65,000	3.5	Gran Canaria	307,545	Wind
Las Palmas IV	15,000	2.61	Gran Canaria	70,972	Wind
BAXTER S.A.	100	3.5	Gran Canaria	200	Photovoltaic
El Corte Inglés, S.A.	300	3.5	Gran Canaria	3000	Photovoltaic
Anfi del Mar I	250	3.5	Gran Canaria	500	Photovoltaic
Anfi del Mar II	250	3.5	Gran Canaria	500	Photovoltaic
AQUALING	2000	3.04	Gran Canaria	4000	Wind
Puerto Rico	4000	3.04	Gran Canaria	8000	Wind
Puerto Rico I	4000	3.04	Gran Canaria	8000	Wind
Hotel Taurito	400	3.04	Gran Canaria	800	Photovoltaic
Hotel Costa Meloneras	300	3.04	Gran Canaria	600	Photovoltaic
Hotel Villa del Conde	500	3.04	Gran Canaria	1000	Photovoltaic
Bahia Feliz	600	3.5	Gran Canaria	1200	Photovoltaic
Bonny	8000	3.5	Gran Canaria	Irrigation	Wind
Maspalomas I Mar	14,500	3.5	Gran Canaria	19,572	Wind
Maspalomas II	25,200	3.04	Gran Canaria	34,016	Wind
UNELCO II	600	3.5	Gran Canaria	Industrial	Photovoltaic
Ayto. San Nicolas	5000	3.04	Gran Canaria	7608	Wind
Asociación de agricultores de la Aldea	5400	3.04	Gran Canaria	Irrigation	Wind
Sureste III	8000	3.5	Gran Canaria	133,846	Wind
Aeropuerto I	1000	3.5	Gran Canaria	24,791	Wind
Salinetas	16,000	3.5	Gran Canaria	102,424	Wind
Aeropuerto II	500	3.5	Gran Canaria	12,396	Photovoltaic
Hoya León	1500	3.5	Gran Canaria	Irrigation	Wind
Bco. García Ruiz	1000	3.5	Gran Canaria	Irrigation	Wind
Mando Aéreo de Canarias	1000	3.5	Gran Canaria	3000	Wind
UNELCO I	1000	3.5	Gran Canaria	Industrial	Wind
Anfi del Mar	1500	3.04	Gran Canaria	3000	Wind
Norcrost. S.A.	170	3.04	Gran Canaria	340	Photovoltaic
Adeje Arona	30,000	3.04	Tenerife	126,728	Wind
Gran Hotel Anthelia Park		3.04	Tenerife	Tourism	Photovoltaic
La Caleta (Ayto. Adeje)	10,000	3.04	Tenerife	20,000	Wind
UTE Tenerife Oeste	14,000	2.16	Tenerife	40,000	Wind
Hotel Sheraton La Caleta		3.04	Tenerife	Tourism	Photovoltaic
Hotel Gran Tacande		3.04	Tenerife	Tourism	Photovoltaic
Hotel Rocas de Nivaria. Playa Paraíso		3.04	Tenerife	Tourism	Photovoltaic
Hotel Bahía del Duque. Costa Adeje		3.04	Tenerife	Tourism	Photovoltaic
Siam Park		3.04	Tenerife	Tourism	Photovoltaic
Tenerife-Sol S. A.		3.04	Tenerife	Tourism	Photovoltaic
Hotel Conquistador, P. de Las Américas		3.04	Tenerife	Tourism	Photovoltaic
Arona Gran Hotel, Los Cristianos		3.04	Tenerife	Tourism	Photovoltaic
Bonny S.A., Finca El Fraile.		3.04	Tenerife	Tourism	Photovoltaic
El Toscal, La Estrella (C. Regantes Las Galletas)		3.04	Tenerife	Tourism	Photovoltaic
Complejo Mare Nostrum, P. Las Américas		3.04	Tenerife	Tourism	Photovoltaic
Hotel Villa Cortés		3.04	Tenerife	Tourism	Photovoltaic
Buenavista Golf, S.A.		3.04	Tenerife	Tourism	Photovoltaic
Rural Teno		3.04	Tenerife	Agrícola	Photovoltaic
Ropa Rent, S.A. (P.I. Güímar)		3.04	Tenerife	Industrial	Photovoltaic
Unelco	600	3.5	Tenerife	Industrial	Photovoltaic
I.T.E.R. Cabildo de Tenerife	14	3.5	Tenerife	Industrial	Photovoltaic
C.T. en P.I. de Granadilla		3.5	Tenerife	Industrial	Photovoltaic
Bonny S.A., Finca El Confital.		3.5	Tenerife	Irrigation	Photovoltaic
Polígono Industrial de Granadilla (portátil)		3.5	Tenerife	Industrial	Photovoltaic
UTE Desalinizadora de Granadilla	14,000	3.04	Tenerife	50,146	Wind
Guia de ISORA Hoya de la leña		3.5	Tenerife	Tourism	Photovoltaic
Club Campo Guía de Isora, Abama		3.5	Tenerife	Tourism	Photovoltaic
Hotel Meliá Palacio de Isora, Alcalá.		3.5	Tenerife	Tourism	Photovoltaic
Loro Parque		3.5	Tenerife	Tourism	Photovoltaic
Santa Cruz I	20,000	3.04	Tenerife	204,856	Wind
Recinto Portuario Santa Cruz (portátil)		3.04	Tenerife	Industrial	Photovoltaic
CEPSA	1000	3.04	Tenerife	Industrial	Wind
Hotel Playa la Arena		3.04	Tenerife	Tourism	Photovoltaic
Hotel Jardín Tecina	2000	3.04	La Gomera	4000	Wind
La Restinga	500	3.5	El Hierro	297	Photovoltaic
La Restinga	1200	3.04	El Hierro	712	Wind
El Cangrejo	1200	3.04	El Hierro	2478	Wind
El Cangrejo	1200	3.04	El Hierro	2478	Wind
El Golfo	1350	3.04	El Hierro	4093	Wind

**Table 7:** Existing seawater desalination plants in the Canary Islands. Source: FCCA 2013 and REE 2020.

Name of the Plant	Production (m^3^/d)	Consume (kWh/m^3^)	Economic Cost (€/m^3^)	Carbon Footprint (tCO_2_/m^3^)	Ecological Footprint (ha/Year/tCO_2_/m^3^)
Cercado de Don Andrés	200	3.5	0.040448289	0.0021	0.00105
Lanzarote III 1	10,000	3.5	0.040448289	0.0021	0.00105
Lanzarote III 2	5000	3.5	0.040448289	0.0021	0.00105
Lanzarote III 3	5000	3.5	0.040448289	0.0021	0.00105
Lanzarote IV	20,000	3.5	0.040448289	0.0021	0.00105
Lanzarote V	18,000	2.4	0.02773597	0.001566	0.00072
Aeropuerto	700	3.04	0.035132228	0.001824	0.000912
Agua Park	30	3.04	0.035132228	0.001824	0.000912
Apartamentos Ficus	60	3.5	0.040448289	0.0021	0.00105
Apartamentos Puerto Tahiche	150	3.5	0.040448289	0.0021	0.00105
Apartamentos Trebol	80	3.5	0.040448289	0.0021	0.00105
Ercros	2500	3.5	0.040448289	0.0021	0.00105
Ercros	2200	3.5	0.040448289	0.0021	0.00105
Famara	350	3.5	0.040448289	0.0021	0.00105
Hotel Golf y Mar	90	3.5	0.040448289	0.0021	0.00105
Hotel Gran Meliá Salinas	400	2.61	0.030162867	0.001566	0.000783
Hotel Playa Verde	250	3.5	0.040448289	0.0021	0.00105
Hotel Teguise Playa	250	3.5	0.040448289	0.0021	0.00105
La Galea	150	3.04	0.035132228	0.001824	0.000912
Lanzarote Beach Club II	70	3.04	0.035132228	0.001824	0.000912
Las Arenas, Costa Teguise	80	3.04	0.035132228	0.001824	0.000912
Playa Roca	250	3.04	0.035132228	0.001824	0.000912
Apartamentos Don Paco Castilla	320	2.61	0.030162867	0.001566	0.000783
Apartamentos Sol Lanzarote	350	2.61	0.030162867	0.001566	0.000783
Cdad Apartamentos CAMP		2.61	0.030162867	0.001566	0.000783
Holiday Land S.A.	3000	3.5	0.040448289	0.0021	0.00105
Hotel Fariones Playa	500	3.5	0.040448289	0.0021	0.00105
Hotel Playa Azul	300	3.5	0.040448289	0.0021	0.00105
Hoteles Canarios S.A.		3.5	0.040448289	0.0021	0.00105
Iberhotel		3.5	0.040448289	0.0021	0.00105
Zorilla	40	3.04	0.035132228	0.001824	0.000912
Hotel Jameos Playa	336	2.61	0.030162867	0.001566	0.000783
La Santa Sport I	250	3.5	0.040448289	0.0021	0.00105
La Santa Sport II	250	3.5	0.040448289	0.0021	0.00105
Ria La Santa	400	3.5	0.040448289	0.0021	0.00105
Apartamentos Son Boy Family Suites	500	3.04	0.035132228	0.001824	0.000912
Bungalows Atlantic Gardens		3.5	0.040448289	0.0021	0.00105
Costa los Limones S.A.	350	3.5	0.040448289	0.0021	0.00105
Hotel Corbeta		3.5	0.040448289	0.0021	0.00105
Hotel Costa Calero	324	3.04	0.035132228	0.001824	0.000912
Marina Rubicón	300	3.04	0.035132228	0.001824	0.000912
Hotel Paradise Island	300	3.04	0.035132228	0.001824	0.000912
Hotel Princesa Yaiza	500	3.04	0.035132228	0.001824	0.000912
Hotel Rubicón Palace	450	3.04	0.035132228	0.001824	0.000912
Inalsa Sur 1	600	3.5	0.040448289	0.0021	0.00105
Inalsa Sur 2	1200	3.5	0.040448289	0.0021	0.00105
Inalsa Sur 3	3000	3.5	0.040448289	0.0021	0.00105
Janubio		3.04	0.035132228	0.001824	0.000912
Lanzasur Club	200	3.04	0.035132228	0.001824	0.000912
Playa Blanca S.A.		3.5	0.040448289	0.0021	0.00105
Club Lanzarote	4500	3.5	0.040448289	0.0021	0.00105
Apartamentos Moromar	250	3.5	0.040448289	0.0021	0.00105
Gea Fonds Numero Uno Lanzarote S.A.		3.5	3.5	0.001623494	0.0021
Grupo Rosa	1000	3.5	0.040448289	0.0021	0.00105
Hipotels	300	3.5	0.040448289	0.0021	0.00105
Hotel Corona	300	3.5	0.040448289	0.0021	0.00105
Hotel Costa Calero S.L.	300	3.04	0.035132228	0.001824	0.000912
Hotel Sunbou	500	3.04	0.035132228	0.001824	0.000912
Isla Lobos	100	3.04	0.035132228	0.001824	0.000912
Leas Hotel S.A.		3.5	0.040448289	0.0021	0.00105
Niels Prahm		3.5	0.040448289	0.0021	0.00105
Occidental Hotel Oasis	250	3.04	0.035132228	0.001824	0.000912
Playa Flamingo	200	3.04	0.035132228	0.001824	0.000912
Tjaereborg Timesharing, S.A.	500	3.04	0.035132228	0.001824	0.000912
Empresa Mixta de Aguas de Antigua, S.L.	4800	3.04	0.035132228	0.001824	0.000912
Grupo Turístico Barceló, S.L.	240	3.5	0.040448289	0.0021	0.00105
Aguas Cristóbal Franquis, S.L.	1200	3.5	0.040448289	0.0021	0.00105
Anjoca Canarias, S.A.	3000	3.5	0.040448289	0.0021	0.00105
Ramiterra, S.L.	3000	3.04	0.035132228	0.001824	0.000912
Inver Canary Dos, S.L.	300	3.04	0.035132228	0.001824	0.000912
Suministros de Agua de La Oliva, S.A.	9000	3.04	0.035132228	0.001824	0.000912
Consorcio Abastecimiento de Aguas a Fuerteventura	4000	3.04	0.035132228	0.001824	0.000912
Parque de Ocio y Cultura BAKU 1	300	3.04	0.035132228	0.001824	0.000912
Parque de Ocio y Cultura BAKU 2	90	3.04	0.035132228	0.001824	0.000912
RIU Palace Tres Islas	100	3.5	0.040448289	0.0021	0.00105
RIU Oliva Beach	400	3.5	0.040448289	0.0021	0.00105
Nombredo, S.L.	500	3.5	0.040448289	0.0021	0.00105
Consorcio Abastecimiento de Aguas a Fuerteventura	4400	3.5	0.040448289	0.0021	0.00105
Puertito de la Cruz	60	3.5	0.040448289	0.0021	0.00105
Vinamar, S.A.	3600	3.5	0.040448289	0.0021	0.00105
Fuercan, S.L. Cañada del Rio I	2000	3.5	0.040448289	0.0021	0.00105
Fuercan, S.L. Cañada del Rio II	1000	3.04	0.035132228	0.001824	0.000912
Fuercan, S.L. Cañada del Rio III	2000	3.04	0.035132228	0.001824	0.000912
Club Aldiana	200	3.5	0.040448289	0.0021	0.00105
Erwin Sick	30	3.5	0.040448289	0.0021	0.00105
Esquinzo Urbanización II	1200	3.5	0.040448289	0.0021	0.00105
Esquinzo Urbanización III	1200	3.5	0.040448289	0.0021	0.00105
Hotel Sol Élite Los Gorriones 1	400	3.5	0.040448289	0.0021	0.00105
Hotel Sol Élite Los Gorriones 2	400	3.5	0.040448289	0.0021	0.00105
Stella Canaris I	300	3.5	0.040448289	0.0021	0.00105
Stella Canaris II	300	3.5	0.040448289	0.0021	0.00105
Stella Canaris III	250	3.5	0.040448289	0.0021	0.00105
Hotel H 10 Playa Esmeralda.	250	3.5	0.040448289	0.0021	0.00105
Hotel “Club Paraíso Playa”	300	3.5	0.040448289	0.0021	0.00105
Urbanización Costa Calma.	110	3.5	0.040448289	0.0021	0.00105
Urbanización Tierra Dorada.	120	3.5	0.040448289	0.0021	0.00105
Zoo-Parque La Lajita.	1300	3.5	0.040448289	0.0021	0.00105
Apartamentos Esmeralda Maris	120	3.5	0.040448289	0.0021	0.00105
Hotel H10 Tindaya	280	3.5	0.040448289	0.0021	0.00105
Aparthotels Morasol	80	3.5	0.040448289	0.0021	0.00105
Consorcio Abastecimiento de Aguas a Fuerteventura	36,500	3.5	0.040448289	0.0021	0.00105
Aeropuerto	500	3.5	0.040448289	0.0021	0.00105
GranTarajal	4000	3.5	0.040448289	0.0021	0.00105
Sotavento, S.A.	2925	3.5	0.040448289	0.0021	0.00105
Arucas-Moya I	10,000	3.5	0.040448289	0.0021	0.00105
Granja experimental	500	3.5	0.040448289	0.0021	0.00105
Granja experimental	500	3.5	0.040448289	0.0021	0.00105
Comunidad Fuentes de Quintanilla	800	3.04	0.035132228	0.001824	0.000912
Granja experimental	500	3.5	0.040448289	0.0021	0.00105
Gáldar-Agaete I	3000	3.5	0.040448289	0.0021	0.00105
Gáldar II	7000	3.04	0.035132228	0.001824	0.000912
Agragua	15,000	3.5	0.040448289	0.0021	0.00105
Guía I	5000	3.5	0.040448289	0.0021	0.00105
Guía II	5000	2.61	0.030162867	0.001566	0.000783
Félix Santiago Melián	5000	2.61	0.030162867	0.001566	0.000783
Las Palmas III	65,000	3.5	0.040448289	0.0021	0.00105
Las Palmas IV	15,000	2.61	0.030162867	0.001566	0.000783
BAXTER S.A.	100	3.5	0.040448289	0.0021	0.00105
El Corte Inglés, S.A.	300	3.5	0.040448289	0.0021	0.00105
Anfi del Mar I	250	3.5	0.040448289	0.0021	0.00105
Anfi del Mar II	250	3.5	0.040448289	0.0021	0.00105
AQUALING	2000	3.04	0.035132228	0.001824	0.000912
Puerto Rico I	4000	3.04	0.035132228	0.001824	0.000912
Puerto Rico II	4000	3.04	0.035132228	0.001824	0.000912
Hotel Taurito	400	3.04	0.035132228	0.001824	0.000912
Hotel Costa Meloneras	300	3.04	0.035132228	0.001824	0.000912
Hotel Villa del Conde	500	3.04	0.035132228	0.001824	0.000912
Bahia Feliz	600	3.5	0.040448289	0.0021	0.00105
Bonny	8000	3.5	0.040448289	0.0021	0.00105
Maspalomas I Mar	14,500	3.5	0.040448289	0.0021	0.00105
Maspalomas II	25,200	3.04	0.035132228	0.001824	0.000912
UNELCO II	600	3.5	0.040448289	0.0021	0.00105
Ayto. San Nicolas	5000	3.04	0.035132228	0.001824	0.000912
Asociación de agricultores de la Aldea	5400	3.04	0.035132228	0.001824	0.000912
Sureste III	8000	3.5	0.040448289	0.0021	0.00105
Aeropuerto I	1000	3.5	0.040448289	0.0021	0.00105
Salinetas	16,000	3.5	0.040448289	0.0021	0.00105
Aeropuerto II	500	3.5	0.040448289	0.0021	0.00105
Hoya León	1500	3.5	0.040448289	0.0021	0.00105
Bco. García Ruiz	1000	3.5	0.040448289	0.0021	0.00105
Mando Aéreo de Canarias	1000	3.5	0.040448289	0.0021	0.00105
UNELCO I	1000	3.5	0.040448289	0.0021	0.00105
Anfi del Mar	1500	3.04	0.035132228	0.001824	0.000912
Norcrost, S.A.	170	3.04	0.035132228	0.001824	0.000912
Adeje Arona	30,000	3.04	0.035132228	0.001824	0.000912
Gran Hotel Anthelia Park		3.04	0.035132228	0.001824	0.000912
La Caleta (Ayto. Adeje)		3.04	0.035132228	0.001824	0.000912
Hotel Sheraton La Caleta		2.16	0.035132228	0.001824	0.000912
Hotel Gran Tacande		3.04	0.035132228	0.001824	0.000912
Hotel Rocas de Nivaria, Playa Paraíso		3.04	3.04	0.00141012	0.001824
Hotel Bahía del Duque, Costa Adeje		3.04	3.04	0.00141012	0.001824
Siam Park		3.04	0.035132228	0.001824	0.000912
Tenerife-Sol S. A.		3.04	0.035132228	0.001824	0.000912
Hotel Conquistador, P. de Las Américas		3.04	3.04	0.00141012	0.001824
Arona Gran Hotel, Los Cristianos		3.04	0.035132228	0.001824	0.000912
Bonny S.A., Finca El Fraile.		3.04	0.035132228	0.001824	0.000912
El Toscal, La Estrella (C. Regantes Las Galletas)		3.04	3.04	0.00141012	0.001824
Complejo Mare Nostrum, P. Las Américas		3.04	3.04	0.00141012	0.001824
Hotel Villa Cortés		3.04	0.035132228	0.001824	0.000912
Buenavista Golf S.A,		3.04	0.035132228	0.001824	0.000912
Rural Teno		3.04	0.035132228	0.001824	0.000912
Ropa Rent, S.A. (P.I. Güímar)		3.04	0.035132228	0.001824	0.000912
Unelco	600	3.04	0.040448289	0.0021	0.00105
I.T.E.R. Cabildo de Tenerife	14	3.5	0.040448289	0.0021	0.00105
C.T. en P.I. de Granadilla		3.5	0.040448289	0.0021	0.00105
Bonny S.A., Finca El Confital.		3.5	0.040448289	0.0021	0.00105
Polígono Industrial de Granadilla (portatil)		3.5	3.5	0.001623494	0.0021
Guia de ISORA Hoya de la leña		3.5	0.040448289	0.0021	0.00105
Club Campo Guía de Isora, Abama		3.5	3.04	0.001623494	0.0021
Hotel Meliá Palacio de Isora, Alcalá		3.5	3.5	0.001623494	0.0021
Loro Parque		3.5	0.040448289	0.0021	0.00105
Santa Cruz I	20,000	3.5	0.035132228	0.001824	0.000912
Recinto Portuario Santa Cruz (portátil)		3,04	3.5	0.00141012	0.001824
CEPSA	1000	3.04	0.035132228	0.001824	0.000912
Hotel Playa la Arena		3.04	0.035132228	0.001824	0.000912
Hotel Jardín Tecina	2000	3.04	0.035132228	0.001824	0.000912
La Restinga	500	3.04	0.040448289	0.0021	0.00105
La Restinga	1200	3.04	0.035132228	0.001824	0.000912
El Cangrejo	1200	3.5	0.035132228	0.001824	0.000912
El Cangrejo	1200	3.04	0.035132228	0.001824	0.000912
El Golfo	1350	3.04	0.035132228	0.001824	0.000912

**Table 8:** Carbon footprint according to the technological structure of the generation parks that uses oil products in the Canary Islands, broken down by islands (2017) [15].

CO_2_ Footprint per Non-Renewable Technology in Canaries (tCO_2_)							
Technology	Gran Canaria	Tenerife	Lanzarote	Fuerteventura	La Palma	La Gomera	El Hierro
Vapor Turbine	274,429	279,289	550,154	34,9597	268,273	68,688.22	48,276
Diesel Motor	110,730	193,188	39,888	50,483	14,360	-	-
Gas Turbine	1,270,058	1,110,153	-	-	-	-	-
Combined Cycle	1,175,213	1,162,741	-	-	-	-	-

**Table 9:** Carbon footprint by installed power according to the technological structure of the generation park that uses oil products in the Canary Islands, broken down by islands (2017) [15].

CO_2_ Carbon Footprint per Power Installed of Non-Renewable Technology in Canaries (tCO_2_/MW)	
Technology	tCO_2_/MW
Vapor Turbine	3240
Diesel Motor	638
Gas Turbine	4175
Combined Cycle	2545

**Table 10:** CO_2_ footprint of each non-renewable technology per MWh in the Canary Islands (tCO_2_/MWh) (2017) [16].

CO_2_ Footprint of Each Non-Renewable Technology per MWh in Canarias (tCO_2_/MWh)							
Technology	Gran Canaria	Tenerife	Lanzarote	Fuerteventura	La Palma	La Gomera	El Hierro
Diesel Motor	0.224621	0.204929	0.151737	0.12329	0.215532	0.168356	0.364811
Gas Turbine	0.178854	0.170368	0.278681	0.045057	2.494326	0	0
Vapor Turbine	0.145261	0.134765	0	0	0	0	0
Combined Cycle	0.175115	0.162457	0	0	0	0	0

**Table 11:** Percentages of water consumption by islands and sectors (%) (2017) [15].

Water Consumes in Canarias per Sectors (%)							
Consume	Lanzarote	Fuerteventura	Gran Canaria	Tenerife	La Gomera	El Hierro	La Palma
**Urban**	26%	29%	32%	27%	9%	23%	8%
**Touristic**	40%	48%	11%	10%	9%	3%	2%
**Industrial**	3%	4%	4%	5%	0%	1%	0%
**Irrigation**	23%	11%	43%	49%	69%	63%	77%
**Losses**	7%	9%	9%	9%	13%	9%	13%
**Total**	100%	100%	100%	100%	100%	100%	100%

## Data Availability

Not applicable.
